# Unveiling the immunomodulatory properties of *Haemonchus contortus* adhesion regulating molecule 1 interacting with goat T cells

**DOI:** 10.1186/s13071-020-04297-7

**Published:** 2020-08-18

**Authors:** Mingmin Lu, Xiaowei Tian, Yang Zhang, Kalibixiati Aimulajiang, Wenjuan Wang, Muhammad Ehsan, Charles Li, Ruofeng Yan, Lixin Xu, Xiaokai Song, Xiangrui Li

**Affiliations:** 1 MOE Joint International Research Laboratory of Animal Health and Food Safety, College of Veterinary Medicine, Nanjing Agricultural University, Nanjing, 210095 Jiangsu People’s Republic of China; 2 Animal Biosciences and Biotechnology Laboratory, Beltsville Agricultural Research Center, Agricultural Research Service, USA Department of Agriculture, Beltsville, MD 20705 USA

**Keywords:** *H. contortus*, Excretory-secretory protein, Adhesion-regulating molecule 1 (ADRM1), Immunomodulation, Immune evasion

## Abstract

**Background:**

Gastrointestinal nematodes could release excretory-secretory (ES) proteins into the host environment to ensure their survival. These ES proteins act as immunomodulators to suppress or subvert the host immune response *via* the impairment of immune cell functions, especially in chronic infections. In our preliminary study, *Haemonchus contortus* adhesion-regulating molecule 1 (HcADRM1) was identified from *H. contortus* ES proteins (HcESPs) that interacted with host T cells *via* liquid chromatography-tandem mass spectrometry analysis. However, little is known about HcADRM1 as an ES protein which may play a pivotal role at the parasite-host interface.

**Methods:**

Based on bioinformatics approaches, multiple amino acid sequence alignment was conducted and the evolutionary relationship of HcADRM1 with ADRM1 orthologues was extrapolated. Employing RT-qPCR and immunohistochemistry assays, temporal transcriptional and spatial expression profiles of HcADRM1 were investigated. Using immunostaining approaches integrated with immunological bioassays, the immunomodulatory potentials of HcADRM1 on goat T cells were assessed.

**Results:**

We hereby demonstrated that HcADRM1 with immunodiagnostic utility was a mammalian ADRM1 orthologue abundantly expressed at all developmental stages of *H. contortus*. Given the implications of ADRM1 proteins in cell growth, survival and development, we further investigated the immunomodulatory property of HcADRM1 as an individual ES protein acting at the parasite-host interface. The rHcADRM1 stimuli notably suppressed T cell viability, promoted intrinsic and extrinsic T cell apoptosis, inhibited T cell proliferation and induced cell cycle arrest at G1 phase. Simultaneously, rHcADRM1 stimuli exerted critical controls on T cell cytokine secretion profiles, predominantly by restraining the secretions of interleukin (IL)-4, IL-10 and interferon-gamma.

**Conclusions:**

Importantly, HcADRM1 protein may have prophylactic potential for anti-*H. contortus* vaccine development. Together, these findings may contribute to the clarification of molecular and immunomodulatory traits of ES proteins, as well as improvement of our understanding of parasite immune evasion mechanism in *H. contortus*-host biology.
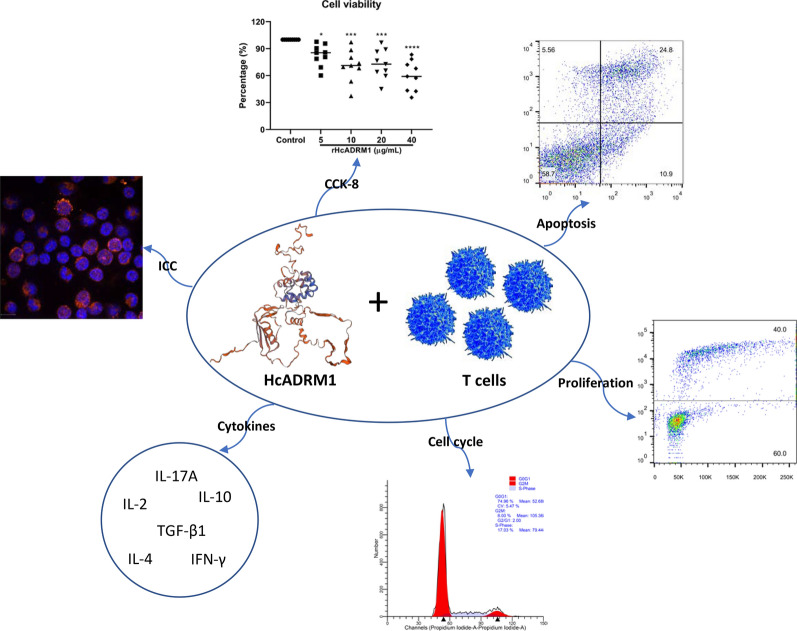

## Background

The highly conserved and regulated ubiquitin (Ub) proteasome pathway is the primary mechanism for targeted elimination of most short-lived proteins including misfolded or damaged proteins in eukaryotic cells [1]. Ub can covalently attach to cellular proteins by Ub modification which is an ATP-dependent process mediated *via* different classes of Ub enzymes [2]. Alongside three families of shuttling factors (Rad23, Dsk2 and Ddi1), three proteasome subunits located in the sub-complex of 26S proteasome, Rpn1, Rpn10 and Rpn13, are demonstrated to be Ub receptors as well. As the proteasome-associated polyubiquitin receptor, Rpn13, also termed as adhesion-regulating molecule 1 (ADRM1), is recruited by Rpn2 to be assembled into the 19S regulatory particle and target protein substrates linked to the small protein Ub *via* its pleckstrin-like receptor [3, 4]. Simultaneously, the C-terminal adaptor domain of ADRM1 serves to bind and activate the deubiquitylase UCHL5/UCH37, and enhance its isopeptidase activity, revealing a mechanism to accelerate Ub chain disassembly [5–7].

With engagement in the Ub proteasome pathway that regulates a broad range of physiological functions, ADRM1 is implicated in multitudinous cellular processes such as cell growth, migration, survival and development, particularly in cancer cells [8]. Recent publications reveal that ADRM1 transcription is consistently elevated in ovarian, colorectal and gastric cancer tissues, and knockdown of ADRM1 expression in both human colon carcinoma and gastric cancer cell lines suppress cell migration and proliferation, and induces cell apoptosis [9–11]. Meanwhile, Fejzo et al. [12] demonstrated that overexpression of ADRM1 in ovarian cancer promoted cell growth and migration, whereas blocking its expression caused cell death. Given the association of amounting ADRM1 expression with the onset and progression of cancers, ADRM1 has been defined as a potential predictive and therapeutic target for clinical therapy [13]. Additionally, comparable expressions of ADRM1 have also been observed in several lymphocyte cell lines as well as endothelial cell lines, and similar physiological roles of ADRM1 are described through its excessive expression in skin endothelial cells that facilitates T lymphocyte adhesion [14].

In a previous study [15], we identified 114 *Haemonchus contortus* excretory-secretory (ES) proteins (HcESPs) that interacted with host T cells *via* liquid chromatography mass spectrometry (LC-MS/MS) analysis. *Haemonchus contortus* ADRM1 (HcADRM1) protein, a mammalian ADRM1 homologue, was ascertained among these interacting proteins [15]. Additionally, recombinant HcADRM1 (rHcADRM1) was recognized by serum samples obtained at Day 7, 14, 21, 35, 49, 63 and 85 post-infection, derived from experimentally *H. contortus*-infected goats. As a result of these observations, HcADRM1 with immunodiagnostic utility was fostered as a hallmark of *H. contortus* infection, and a serological diagnosis assay with high sensitivity and specificity was developed using HcADRM1 antigen [16]. Furthermore, our preliminary analysis showed that HcESPs stimuli notably induced intrinsic and extrinsic apoptosis, suppressed T cell proliferation, and caused cell cycle arrested. HcESPs consisted of multitudinous modulatory molecules such as kinases, phosphatases, hydrolases and proteases, where the pleiotropic effects were initiated by a cascade of individual ES components. Importantly, the exact molecules that modulated T cell immune response in the parasite-host interaction warrant further investigation. Given the functional diversity of ADRM1, and especially its engagement in cell proliferation and apoptosis, HcADRM1 might be one of these dominated proteins that exert critical controls on cellular survival and death of host key effector cells. Therefore, herein we aimed to further investigate the molecular traits of HcADRM1 and address its immunomodulatory roles at the parasite-host interface.

## Methods

### Parasite, animals and cells

The *H. contortus* strain was propagated *via* serial passages in nematode-free goats in the Animal Experimental Center, Faculty of Veterinary Medicine, Nanjing, China. The collection of eggs, L3, xL3, male and female adults was performed as previously described [17, 18]. Sprague Dawley (SD) rats (SCXK 2008-0004) with a standard packing weight (~ 150 g) were obtained from Jiangsu Experimental Animal Center (Nanjing, China). They were maintained in a microbe-free room with access to sterilized food and water *ad libitum*.

Local crossbred and healthy goats (5–6 months-old) were reared in individually ventilated cages to prevent accidental infection with nematodes. Alongside *ad libitum* access to water in pens, these goats were given hay and whole shelled corn daily. Peripheral venous blood samples were obtained by venipuncture as described elsewhere, as well as the isolation of goat peripheral blood mononuclear cells (PBMCs) [19]. Total T cells in goat PBMCs were sorted using a magnetic-activated cell sorting system (MACS; Miltenyi Biotech Inc, Auburn, CA, USA) as described elsewhere [20]. Briefly, every million PBMCs in 100 µl staining buffer were incubated with 10 µl mouse anti-bovine CD2 primary antibody (Bio-Rad, Kidlington, UK) which cross-react with goat CD2 T cells for 30 min. After two washes in PBS, 1 × 10^7^ of total PBMCs resuspended in 100 µl staining buffer were labeled with 10 µl anti-FITC MicroBeads (Miltenyi Biotech) at room temperature for 15 min. Subsequently, PBMCs were loaded onto the MACS magnetic system (Miltenyi Biotech) for positive sorting based on the manufacturer’s specifications. T cells were then adjusted to a density of 1 × 10^6^ cells/ml in RPMI 1640 (Gibco, Grand Island, NY, USA) containing 10% heat-inactivated fetal calf serum (FCS; Gibco) and 100 U/ml penicillin, 100 mg/ml streptomycin (Gibco). The viability of T cells was > 95% as assessed by the trypan blue exclusion test. The 95% purity of separated T cells was validated *via* flow cytometric determination (Additional file 1: Figure S1). Three goats (biological replicates) were used in every experiment.

### Sequence alignment and phylogenetic analysis

The cloning and amplification of the complete coding sequence of the *HcADRM1* gene were performed as previously described [16]. The amplified *HcADRM1* fragment was cloned into pET28a (+) vector (Invitrogen, Carlsbad, CA, USA) and validated by sequence analysis using BLAST. Multiple amino acid sequences of ADRM1 orthologs were aligned for comparison by the CLUSTAL OMEGA software [21]. The evolutionary analysis was conducted and extrapolated by MEGA X software using JTT matrix-based model and the Maximum Likelihood method [22]. With partial deletion option, positions with less than 80% site coverage were excluded prior to phylogenetic analysis. Based on the optimized evaluation of 1000 replicates for bootstrap support, the evolutionary tree of ADRM1 orthologues was generated with several designated and collapsed branches.

### Expression and purification of the rHcADRM1 protein

The expression and purification of rHcADRM1 proteins were conducted as elsewhere described [23]. Briefly, *Escherichia coli* BL21 (DE3) cells containing the reconstructed pET28a-HcADRM1 plasmid were incubated with Luria-Bertini medium containing kanamycin (100 µg/ml; Sigma-Aldrich, St. Louis, MO, USA) and then stimulated by isopropyl-β-d-thiogalactopyranoside (Sigma-Aldrich) for the induction of rHcADRM1 expression. The rHcADRM1 protein fused with a histidine-tag was obtained from the supernatant of cell lysates *via* His-Trap HP purification columns (GE Healthcare, Piscataway, NJ, USA). rHcADRM1 proteins were resolved on 12% sodium dodecyl sulfate-polyacrylamide gel electrophoresis (SDS-PAGE) gels for size and purity validation, and the concentration was determined by a bicinchoninic acid (BCA) assay (Thermo Fisher Scientific, Rockford, IL, USA). Employing the Detoxi-Gel Affinity Pak prepacked columns (Thermo Fisher Scientific), rHcADRM1 proteins were exempt of lipopolysaccharide contamination. As rHcADRM1 protein was dissolved in PBS, PBS-treated T cells served as the control group (0 µg/ml) in functional assays. The purified rHcADRM1 was stored at − 80 °C until further analysis.

### Preparation of polyclonal antibody (pAb)

To obtain antigen-specific pAb, rHcADRM1 proteins (300 µg) blended with Freund’s complete adjuvant was administrated subcutaneously into SD rats. With a 2-week interval, booster immunizations with 300 µg of rHcADRM1 proteins emulsified in Freund’s incomplete adjuvant were administered four times. Seven days after the final boost, rat sera containing anti-rHcADRM1 pAb were harvested and kept at − 80 °C for further analysis. The sera harvested from *H. contortus*-infected goats (anti-*H. contortus* serum) were stored at the Veterinary Parasitology Teaching and Research Center of Nanjing Agricultural University, Nanjing, China.

### Immunoblot analysis

rHcADRM1 and HcESPs were resolved on protein gels, respectively, and transferred onto nitrocellulose membranes. The blots were blocked using 4% BSA in TRIS-buffered saline-0.1% Tween 20 (TBST) for 1 h at room temperature. The blots with the rHcADRM1 samples were probed with primary goat anti-*H. contortus* serum (1:500 in TBST) or normal goat serum (control) at 4 °C overnight, while the blots with the HcESPs samples were probed with primary rat anti-rHcADRM1 IgG (1:500 in TBST) or normal rat IgG (control). After five washes in TBST, the blots were incubated with horseradish peroxidase-coupled rabbit anti-goat or anti-rat IgG (H+L) secondary antibody (Sigma-Aldrich) in TBST (1:5000) for 1 h at 37 °C. The blots were then developed with 3,3′-diaminobenzidine (DAB; Sigma-Aldrich) for 3–5 min and visualized by using a ChemiDoc imaging system (Bio-Rad, Hercules, CA, USA).

### HcADRM1 transcription in *H. contortus* life-cycle stages

To detect mRNA expression of HcADRM1 in *H. contortus* life-cycle stages, total RNA of eggs, L3, xL3, female and male adults were extracted using Trizol (Invitrogen), and the resulting cDNAs were synthesized in accordance with the manufacturer’s specifications. Employing specific primers for the β-tubulin gene (endogenous reference) [24] and target gene *HcADRM1* (Additional file 2: Table S1), transcriptional analysis of the *HcADRM1* gene was conducted by real-time PCR using the QuantStudio 3 System (Applied Biosystems, Carlsbad, CA, USA) with a standard protocol. The specificity of the primers was validated to ensure product purity *via* generation of a melt curve and the absence of primer dimers. The amplification efficiencies and correlation coefficients were verified to be stable and similar. Based on the 2^−ΔΔCq^ method, the relative transcription levels of HcADRM1 were normalized on β-tubulin transcription. Each experiment was run in triplicate.

### Immunohistochemistry assays

Freshly collected female and male adults were washed, dehydrated, fixed, embedded and cut into cryostat sections as previous described [25]. To minimize non-specific binding, cryosections were treated with 10% normal goat serum in PBS containing 0.1% Tween 20 (PBST) for 1 h. Subsequently, cryosections were served with primary anti-rHcADRM1 IgG (1:200) or sham control IgG overnight at 4 °C. Prior to DNA staining with 2-(4-Amidinophenyl)-6-indolecarbamidine dihydrochloride (DAPI; Sigma-Aldrich), cryosections were then incubated with Cy3-labeled goat anti-rat IgG (1:500; Beyotime Biotechnology, Shanghai, China) at 37 °C for 1 h. Subsequently, the samples were immersed in anti-fade medium (Sigma-Aldrich) to prevent fluorescence fading for microscopic examination. Finally, the sections were imaged at 60× magnification using a LSM710 fluorescence microscope (Zeiss, Jena, Germany), and ZEN 2012 software (Zeiss) was used for the analysis of digital images.

### The interaction of HcADRM1 protein with T cells *in vitro*

The interaction of HcADRM1 with goat T cells was investigated as previously described [26]. In brief, freshly sorted T cells were cultured with or without 5 µg/ml rHcADRM1 proteins for 2 h at 37 °C. After three washes, 4% paraformaldehyde-fixed T cells were permeabilized by 0.5% Triton X-100 in PBST and blocked with 4% BSA in PBST for 30 min. Subsequently, prior to the staining with the Cy3-coupled secondary antibody (1:500), T cells were treated by primary anti-HcADRM1 pAb (1:100) or normal rat IgG (control) in a humidified chamber at 37 °C for 1 h. Followed by five PBST washes, T cells were subjected to Gold Anti-fade mounting solution containing DAPI (Life Technologies, Eugene, OR, USA) for nuclear staining. Immunofluorescence-labeled cells were visualized at 100× magnification using a LSM780 confocal microscope (Zeiss, Jena, Germany); Zen 2012 software (Zeiss) was employed for the interpretation of digital images.

### Cell viability

The modulatory effects of rHcADRM1 on goat T cell viability were determined using the cell counting kit-8 assay (CCK-8; Dojindo, Kumamoto, Japan) as previously described [27]. Fresh sorted goat T cells activated with concanavalin A (ConA, 5 µg/ml) were incubated in the presence of various doses of rHcADRM1 proteins (0, 5, 10, 20 and 40 µg/ml) at 37 °C. Following 24 h-stimulation, cell culture medium was incorporated with 10 µl CCK-8 solution and incubated at 37 °C in the dark for 4 h. Following incubation, optical density was measured at 450 nm (OD450) using a microplate reader (Bio-Rad, Hercules, California, USA). Three independent tests, each in triplicate, were performed.

### Cell apoptosis assay

Flow cytometry assays were performed for T cell apoptosis determination using the Annexin V-PE kit (BD Biosciences, San Jose, CA, USA) as previously described [28]. In brief, freshly sorted T cells were cultured in the presence of tested doses of rHcADRM1 proteins (0, 5, 10, 20 and 40 µg/ml) followed by Annexin V and 7-aminoactinomycin D (7-AAD) staining based on the kit’s specification. The PBS-stimulated T cells served as negative controls. Three individual tests, each in triplicate, were conducted.

### Cell proliferation assay

Cell proliferation analysis was determined using the Alexa Fluor 647 Click-iT plus EdU flow cytometry kit (Thermo Fisher Scientific) *via* the measurement of DNA synthesis directly based on the manufacturer’s instructions. After 12 h co-incubation, the cell culture was incorporated with 5-ethynyl-2′-deoxyuridine (EdU, 10 μM) for another 12 h incubation. Subsequently, T cells were harvested, fixed with 4% paraformaldehyde in PBS and permeabilized using the Click-iT saponin-based reagent, followed by Click-iT reaction to coupled EdU with Alexa Fluor 647 dye. After three washes with 3 ml of 1% BSA in PBS, T cells were treated with 7-AAD staining solution (BD Biosciences). Flow cytometry was used for the determination of EdU^+^ cells in the population. Each experiment consisting of three replicates was run in triplicate.

### Cell cycle assay

Flow cytometry assays were conducted for cell cycle determination using PI/RNase staining buffer (BD Biosciences) according to the manufacturer’s DNA staining protocol. Following co-incubation with rHcADRM1 stimuli (20 µg/ml) for 24 h, T cells were harvested, washed and fixed with ice-cold 75% ethanol every 6 h. After being frozen at − 20 °C for more than 2 h, treated-T cells were washed twice with PBS to remove remaining ethanol and resuspended in PI/RNase staining buffer for flow cytometry analysis. Each experiment consisting of three replicates was run in triplicate.

### Transcription analysis

T cells treated with different concentrations of rHcADRM1 (0, 5, 10, 20 and 40 µg/ml) for 12 h were harvested for the transcription analysis of the cell apoptosis pathway, and T cells treated with 20 µg/ml of rHcADRM1 for 24 h were collected for transcription analysis of the cell cycle pathway. Cells were harvested for total RNA extraction and cDNA obtained by reverse-transcription PCR. Relative quantification of candidate gene expression was conducted using previously published primers [29–33] of endogenous reference and candidate genes (Additional file 2: Table S2). Based on the 2^−ΔΔCq^ method, the relative levels of target gene transcription were normalized to reference gene expression. Each experiment consisting of three replicates was run in triplicate.

### Detection of cytokine secretions

For the determination of cytokine secretion levels, freshly isolated T cells activated by ConA (5 µg/ml) were treated with or without rHcADRM1 (0, 5, 10, 20 and 40 µg/ml) for 24 h. Cell culture medium was harvested and determined for cytokine secretion detection using goat enzyme-linked immunosorbent assay (ELISA) kits (Mlbio, Shanghai, China) according to the manufacturer’s specifications. The limit of quantification dependent upon each analytic kit ranged from between 2 and 800 pg/ml. Each experiment was run in triplicate.

### Statistical analysis

One-way and two-way analysis of variance (ANOVA) with Dunnett’s multiple comparison test, alongside the Student’s t-test, were performed for statistical analysis using GraphPad Premier 8.0 software (GraphPad Prism, San Diego, CA, USA). Differences were regarded as statistically significant when *P*-values were < 0.05. Data were denoted as minimum to maximum (all points) or mean ± standard deviation (SD).

## Results

### Sequence alignment and phylogenetic analysis

**Fig. 1:**
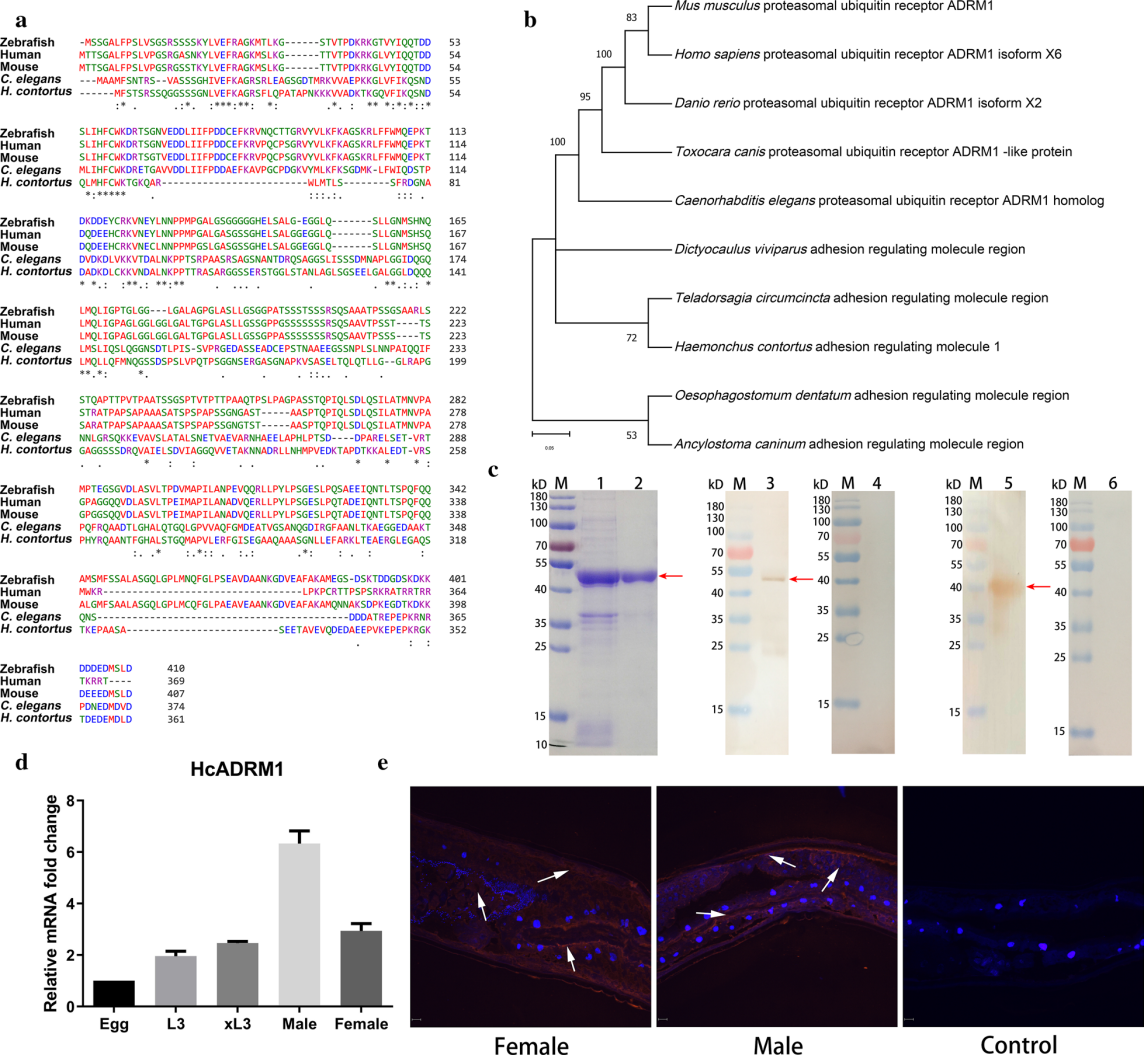
Molecular characterization of HcADRM1 derived from HcESPs. **a** Alignment of HcADRM1 amino acid sequences with other orthologues. The positions of ADRM1 family motifs are indicated above the multiple sequence alignment, containing zebrafish (XP_021325529.1), human (NP_001268366.1), mouse (NP_062796.2), *C. elegans* (NP_498387.2) and *H. contortus* (W6NB91) ADRM1 ortholog sequences retrieved from GenBank. An asterisk indicates the position with one completely conserved amino acid, while period denotes weakly conserved similarity within different groups and colon represents strongly similar conservation between groups. **b** Phylogenetic analysis of HcADRM1 with vertebrate and parasite orthologues. Evolutionary relationships of taxa were inferred using the Maximum Likelihood method with protein sequences including *Mus musculus* (NP_062796.2), *Homo sapiens* (XP_011526805.1), *Danio rerio* (XP_021325529.1), *Toxocara canis* (KHN87202.1), *C. elegans* (NP_498387.2), *Dictyocaulus viviparus* (KJH49054.1), *T. circumcincta* (PIO73930.1), *H. contortus* (W6NB91), *Oesophagostomum dentatum* (KHJ97330.1) and *Ancylostoma caninum* (RCN48176.1). Bootstrap support values are shown for each node. The scale-bar denotes the number of substitutions per site. **c** Acquisition of rHcADRM1 proteins and western blot analysis. Lanes M: protein standard ladder; Lane 1: rHcADRM1 expressed in the supernatant of cell lysates; Lane 2: SDS-PAGE analysis of purified rHcADRM1 protein; Lane 3: immunoblot analysis of rHcADRM1 using anti-*H. contortus* serum as primary antibody; Lane 4: immunoblot analysis of rHcADRM1 using normal goat serum (control) as primary antibody; Lane 5: immunoblot analysis of HcESPs using rat anti-rHcADRM1 IgG as primary antibody; Lane 6: immunoblot analysis of HcESPs using normal rat IgG (control) as primary antibody. **d** HcADRM1 expression in *H. contortus* life-cycle stages. Data are presented as the mean ± SD. **e** Immunolocalization of native HcADRM1 protein in male and female adults. The immunohistochemistry assays were performed using normal rat IgG (control) or rat anti-rHcADRM1 IgG as primary antibody. Cy3-coupled fluorescence (red), along with DAPI (blue), was identified for the investigation of HcADRM1 distribution. *Scale-bars*: **e**, 200 µm

The entire coding region of the *HcADRM1* gene (1083 bp) was amplified from the cDNA of adult worms, encoding a 361-amino acid protein with an estimated molecular mass around 42 kDa. We then performed a sequence alignment of ADRM1 orthologues derived from GenBank on zebrafish, human, mouse, *Caenorhabditis elegans* and *H. contortus* using CLUSTAL OMEGA software. HcADRM1 protein showed a moderate degree of identity to zebrafish (44.79%), human (44.69%), mouse (46.15%) and *C. elegans* (47.14%) orthologs (Fig. 1a). In addition, we conducted an evolutionary analysis of HcADRM1 using the Maximum Likelihood method involving 10 amino acid sequences. Phylogenetic analysis clearly showed an evolutionary relationship of HcADRM1 with other ADRM1 orthologues, revealing that HcADRM1 was closely related to the *Teladorsagia circumcincta* homologue but divergent from vertebrate sequences (Fig. 1b).

### Protein expression and immuno-blot analysis

The rHcADRM1 protein fused with the histidine-tag was successfully obtained in the supernatant of cell lysates. After purification, rHcADRM1 was visualized by Coomassie Blue staining as a single band with a molecular weight of ~ 46 kDa (Fig. 1c; Lane 1). The specificity of the rHcADRM1 protein was determined by western blot, probing with anti-*H. contortus* serum or normal goat serum. A single band ~ 46 kDa was observed through the specific recognition of rHcADRM1 protein by anti-*H. contortus* serum (Fig. 1c; Lane 2), while no band was identified *via* healthy goat sera (Fig. 1c; Lane 3). Meanwhile, native HcADRM1 protein derived from HcESPs was identified by rat anti-rHcADRM1 IgG as a single band of ~ 42 kDa (Fig. 1c; Lane 4), while no positive band was observed in the control groups (Fig. 1c; Lane 5).

### Differential mRNA expression in *H. contortus* life-cycle stages and immunolocalization

Transcription analysis by real-time RT-PCR revealed that mRNA expression of HcADRM1 were detectable at all of the tested *H. contortus* life-cycle stages. The data demonstrated rising expression levels from the free-living stages (eggs and L3) to parasitic stages (xL3, female and male adults). Simultaneously, the highest level of HcADRM1 transcription was observed in male adults, but not in female adults (Fig. 1d). Given that the highest mRNA expression was detected in adult worms, we next investigated the localization of native HcADRM1 proteins within *H. contortus* by checking the cryosections of the adult worms. Specific red fluorescence resulting from tagging HcADRM1 proteins by rat anti-rHcADRM1 IgG was ubiquitously observed from the intracellular and cytoplasmic localization of somatic cells, particularly in the intestinal regions and the internal membrane of cuticle for both male and female adults (Fig. 1e). However, no Cy3-fluorescence was observed in the sections treated with normal rat IgG (Fig. 1e).

### Binding of rHcADRM1 protein to goat T cells

**Fig. 2:**
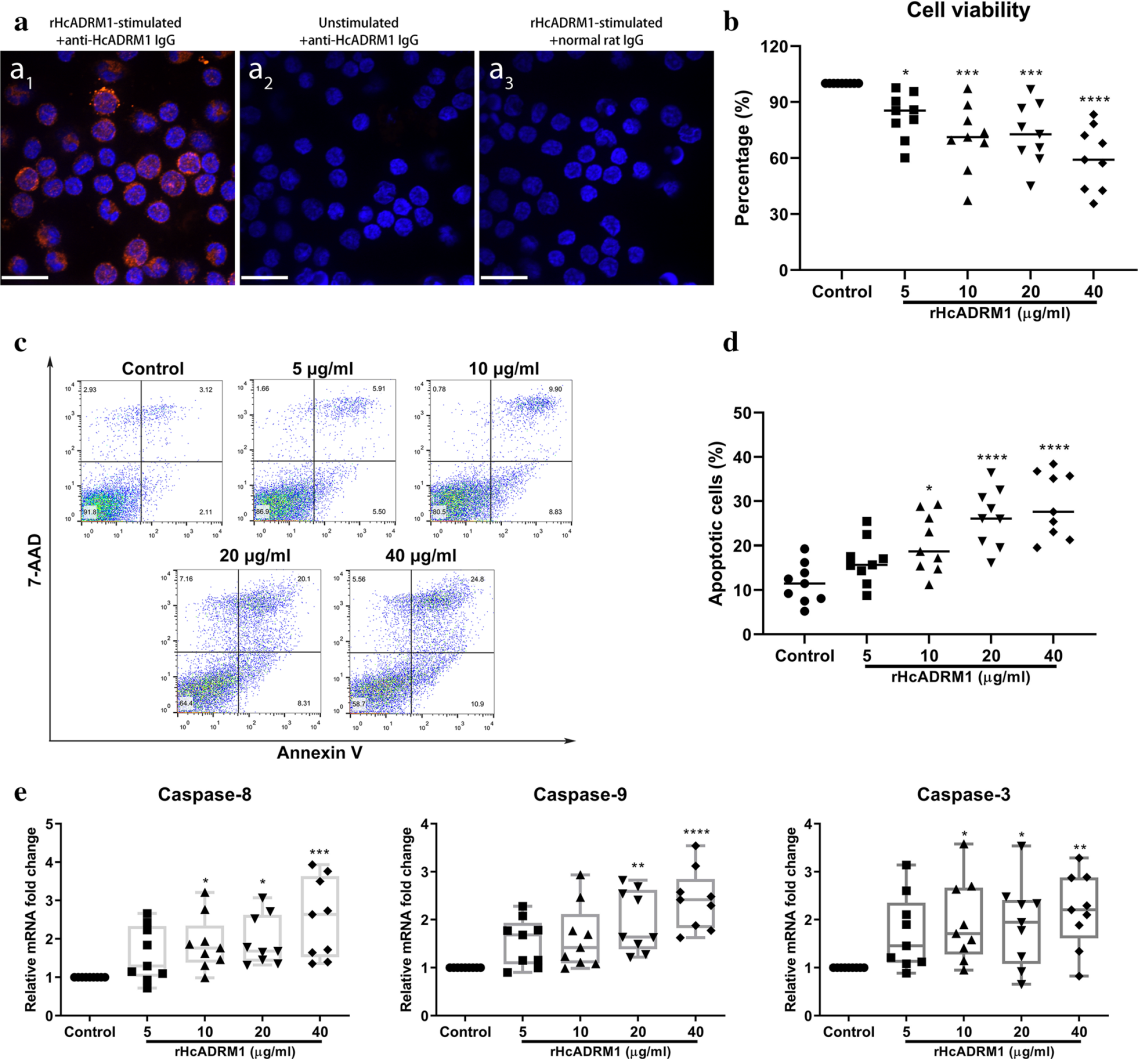
rHcADRM1 proteins suppressed cell viability and induced apoptosis *via* the interaction with goat T cells. **a** Determination of the interaction of HcADRM1 protein with goat T cells *in vitro*. T cells treated with (a_1_) or without (a_2_) rHcADRM1 protein were incubated with rat anti-rHcADRM1 IgG as the primary antibody. T cells stimulated by rHcADRM1 were incubated with normal rat IgG as the primary antibody (a_3_). **b** rHcADRM1 significantly inhibited T cell viability. Cell viability was determined *via* the incorporation with CCK-8, whereas cell viability index was determined by calculating the OD 450 values of the control group as 100%. Results are presented as the mean ± SD. Asterisks denote statistically significant differences (*P < 0.05, ***P < 0.001, ****P < 0.0001) compared with the control group. **c** Flow cytometry analysis of T cell apoptosis in responses to rHcADRM1 stimuli. Apoptosis determination was performed *via* 7-AAD and Annexin V-PE staining. **d** Statistical analysis of T cell apoptosis. The apoptotic proportions were counted *via* the AnnexinV^+^7AAD^−^ and AnnexinV^+^7AAD^+^ T cells. Data are presented as the mean ± SD, where an asterisk denotes a statistically significant difference (*P < 0.05, ****P < 0.0001) compared with the control group. Each experiment was performed in triplicate. **e** The mRNA transcripts of caspase-8, -9 and -3 in rHcADRM1-stimulated T cells. Results are denoted as minimum to maximum (all points). Asterisks denote statistically significant differences (*P < 0.05, **P < 0.01, ***P < 0.001, ****P < 0.0001) compared with the control group. Each experiment was run in triplicate. *Scale-bars*: **a** 100 µm

Based on our preliminary LC-MS/MS analysis, we next conducted immunocytochemistry assays to verify the *in vitro* interaction of HcADRM1 proteins with goat T cells. Immunocytochemistry assay showed that intense red Cy3-fluorescence (resulting from tagging rHcADRM1) was observed in rHcADRM1-treated T cells, revealing the cytomembrane and cytoplasmic localization of rHcADRM1 (Fig. 2a, a_1_); no red fluorescence was detected in both blank and negative control groups (Fig. 2a, a_2_ and a_3_). The results presented here further validated the positive interactions between HcADRM1 protein and host T cells.

### rHcADRM1 suppressed cell viability and induced cell apoptosis

Given the modulatory potential of ADRM proteins on cellular development and survival, we next investigated the impact of rHcADRM1 proteins on T cell viability. The results of CCK-8 determination showed that T cell viability was dramatically inhibited by the stimulation of 5 µg/ml (ANOVA: *F*_(4, 40)_ = 10.01, *P* = 0.0045), 10 µg/ml (ANOVA: *F*_(4, 40)_ = 10.01, *P* = 0.0004), 20 µg/ml (ANOVA: *F*_(4, 40)_ = 10.01, *P* = 0.0009) and 40 µg/ml (ANOVA: *F*_(4, 40)_ = 10.01, *P* < 0.0001) of rHcADRM1 proteins (Fig. 2b). Based on this finding, an Annexin V-PE/7-AAD double staining kit was employed to evaluate the pro-apoptotic potential of rHcADRM1 proteins. Flow cytometry results demonstrated that rHcADRM1 stimuli, at the tested concentrations of 10 µg/ml (ANOVA: *F*_(4, 40)_ = 12.50, *P* = 0.0124), 20 µg/ml (ANOVA: *F*_(4, 40)_ = 12.50, *P* < 0.0001) and 40 µg/ml (ANOVA: *F*_(4, 40)_ = 12.50, *P* < 0.0001) remarkably caused T cell apoptosis in comparison to the unstimulated group (Fig. 2c, d). Additionally, transcriptional analysis of key genes involved in apoptosis signaling pathways further validated the pro-apoptotic effects of rHcADRM1 proteins on host T cells. The treatments with 10, 20 and 40 µg/ml of rHcADRM1 dramatically upregulated mRNA transcripts of caspase-8 (ANOVA: *F*_(4, 40)_ = 5.557, *P* = 0.0409, *P* = 0.0231 and *P* = 0.0002, respectively) and caspase-3 (ANOVA: *F*_(4, 40)_ = 3.296, *P* = 0.0315, *P* = 0.0418 and *P* = 0.0054, respectively) (Fig. 2e). Simultaneously, 20 µg/ml (ANOVA: *F*_(4, 40)_ = 8.265, *P* = 0.0020) and 40 µg/ml (ANOVA: *F*_(4, 40)_ = 8.265, *P* < 0.0001) of rHcADRM1 proteins significantly promoted caspase-9 transcription (Fig. 2e).

### rHcADRM1 protein restrained the proliferation of T cells and caused cell cycle stalling

**Fig. 3:**
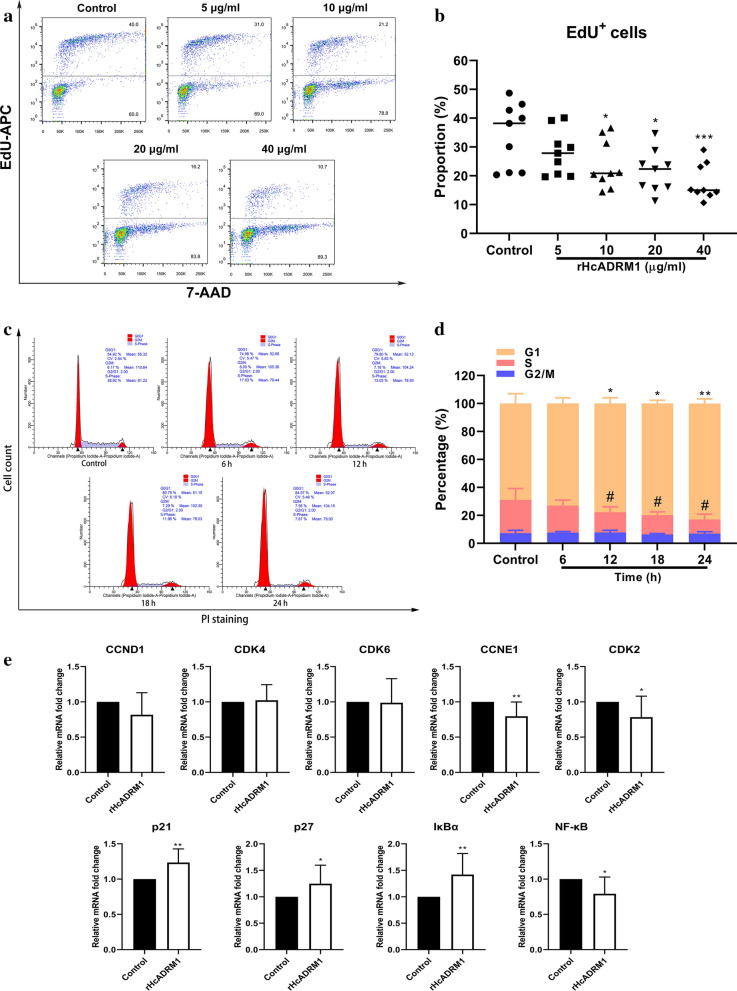
rHcADRM1 protein suppressed T cell proliferation and induced T cell cycle stalling at the G1 phase. **a** Determination of T cell proliferation in responses to rHcADRM1 stimuli. Flow cytometry analysis was performed using 7-AAD and EdU-APC double staining. **b** Statistical analysis of the T cell proliferation rates expressed as the proportions of EdU^+^ T cells. Data are presented as the mean ± SD. Asterisks denote statistically significant differences (*P < 0.05, ***P < 0.001) compared with the control group. **c** T cell cycle analysis in responses to rHcADRM1 stimuli. T cell cycle determination was performed using PI/RNase staining reagent by flow cytometry. **d** Statistical analysis of T cell cycle analysis. T cells incubated with rHcADRM1 proteins were collected every 6 h for flow cytometry analysis. The data demonstrated that rHcADRM1 stimuli caused cell cycle stalling at the G1 phase. Results are presented as the mean ± SD. Asterisks for G1 phase determination (*P < 0.05, **P < 0.01) and ^*#*^ (hashtags) for S phase determination (*P* < 0.05) denote statistically significant differences compared with the control group. Each experiment was run in triplicate. **e** Relative transcription levels of CCND1, CCNE1, CDK4/6, CDK2, p21, p27, IκBα and NF-κB in rHcADRM1-treated T cells. Data are presented as the mean ± SD. Asterisks indicate statistically significant differences (*P < 0.05, **P < 0.01) compared with the control group. Each experiment was run in triplicate

As apoptosis, proliferation and cell cycle were interconnected cellular movements, we next explored the modulatory potentials of rHcADRM1 stimuli on T cell proliferation and cell cycle. At the tested doses of 10, 20 and 40 µg/ml, flow cytometry data showed that rHcADRM1 stimuli significantly inhibited T cell proliferation *in vitro* (Fig. 3a), as indicated by the decreasing proportion of EdU^+^ cells compared with control cells (ANOVA: *F*_(4, 40)_ = 5.150, *P* = 0.0482, *P* = 0.0104 and *P* = 0.0006, respectively) (Fig. 3b). Given that the treatments with 20 µg/ml rHcADRM1 had significant biological effects on cell viability, apoptosis and proliferation, as well as the transcription of certain key genes, we next treated T cells with 20 µg/ml of rHcADRM1 for cell cycle determination. Here, flow cytometry analysis with PI staining demonstrated that rHcADRM1 stimuli induced cell cycle arrest in a time-dependent manner (Fig. 3c), as indicated by the increased proportion of T cells in G1 phase at 12 h (ANOVA: *F*_(8, 96)_ = 17.49, *P* = 0.0293), 18 h (ANOVA: *F*_(8, 96)_ = 17.49, *P* = 0.0165) and 24 h (ANOVA: *F*_(8, 96)_ = 17.49, *P* = 0.0028), as well as the decreased proportion of T cells in S phase at 12 h (ANOVA: *F*_(8, 96)_ = 17.49, *P* = 0.0303), 18 h (ANOVA: *F*_(8, 96)_ = 17.49, *P* = 0.0480) and 24 h (ANOVA: *F*_(8, 96)_ = 17.49, *P* = 0.0118) (Fig. 3d). Consistent with these findings, transcriptional analysis of key genes in G1/S checkpoints showed that mRNA transcripts of CCNE1 (t-test: *t*_(16)_ = 3.030, *P* = 0.0080) and CDK2 (t-test: *t*_(16)_ = 2.180, *P* = 0.0445) were significantly downregulated by rHcADRM1 stimuli, while no significant transcriptional changes of CCND1 (t-test: *t*_(16)_ = 1.748, *P* = 0.0997), CDK4 (t-test: *t*_(16)_ = 0.3238, *P* = 0.7503) and CDK6 (t-test: *t*_(16)_ = 0.1030, *P* = 0.9192) were observed (Fig. 3e). Given the inhibitory effects of p21 and p27 on CDKs in Ub-mediated cell cycle progression, the transcription analysis of p21 and p27 was performed in this study. In addition, the mRNA transcripts of IκBα as the physiological substrate of ADRM1 and its downstream inhibitor NF-κB were determined. Importantly, transcription of p21 (t-test: *t*_(16)_ = 3.665, *P* = 0.0021), p27 (t-test: *t*_(16)_ = 2.131, *P* = 0.0490) and IκBα (t-test: *t*_(16)_ = 3.154, *P* = 0.0061) was notably enhanced by rHcADRM1 stimuli (Fig. 3e), whereas the mRNA transcript of NF-κB (t-test: *t*_(16)_ = 2.623, *P* = 0.0185) was significantly suppressed (Fig. 3e).

### Determination of cytokine secretions

**Fig. 4:**
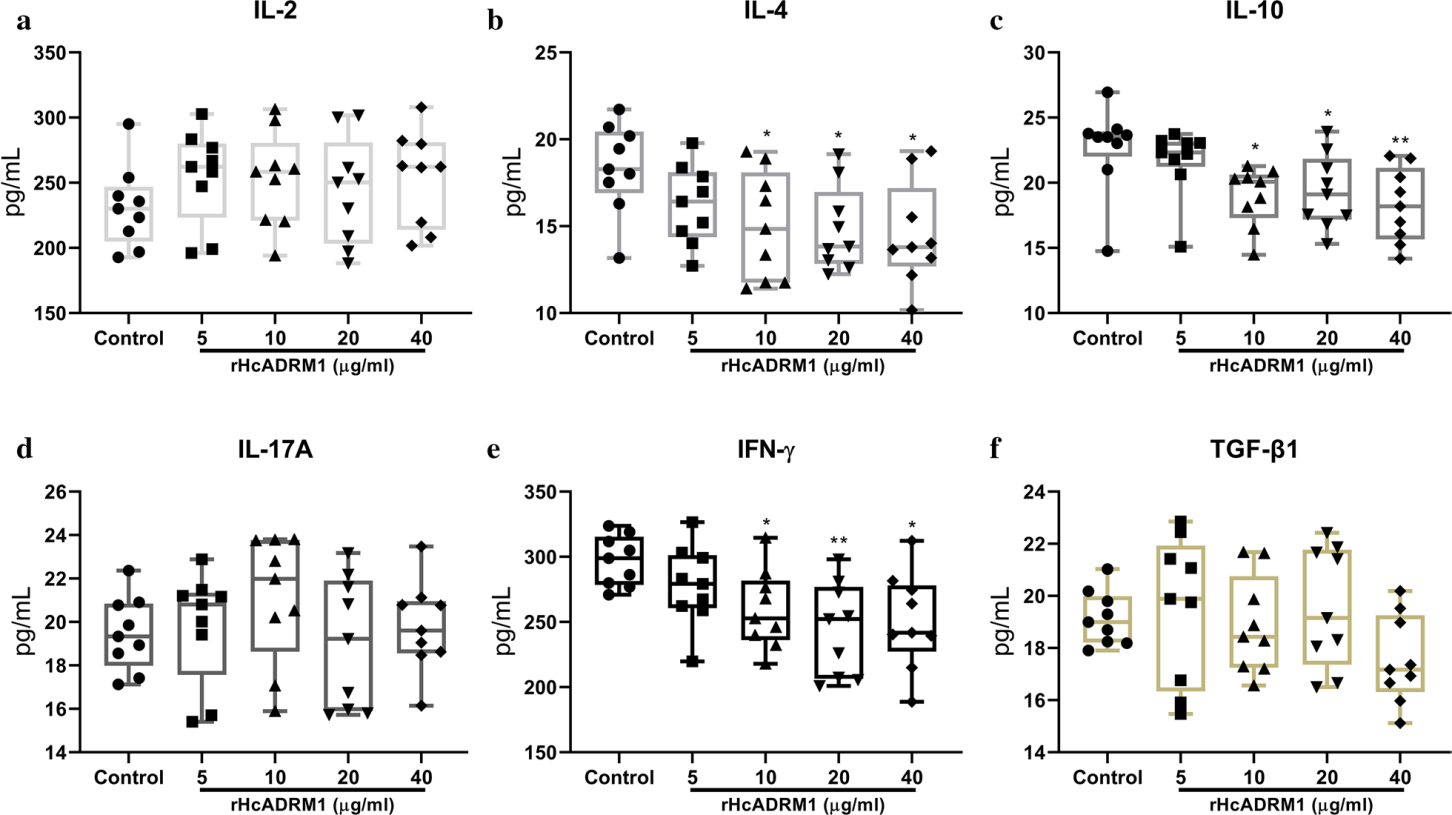
rHcADRM1 stimuli altered T cells cytokine secretion profiles *in vitro*. Goat T cells were stimulated with or without rHcADRM1 proteins for 24 h. The secretions of IL-2 (**a**), IL-4 (**b**), IL-10 (**c**), IL-17A (**d**), IFN-γ (**e**) and TGF-β1 (**f**) in the culture supernatant were determined by ELISA. Data are presented as minimum to maximum (all points). Asterisks denote statistically significant differences (*P < 0.05, **P < 0.01) compared with the control group. Three independent experiments were performed

To investigate the modulatory effects of rHcADRM1 on T cell cytokine productions, IL-2, IL-4, IL-10, IL-17A, IFN-γ and TGF-β1 secretions in the cell culture supernatant were determined *via* ELISA assays. The data showed that the exposure of goat T cells to rHcADRM1 proteins led to the alteration of their cytokine production profiles. Intriguingly, at the tested doses of 10, 20 and 40 µg/ml, rHcADRM1 stimuli predominantly inhibited secretions of IL-4 (ANOVA: *F*_(4, 40)_ = 3.097, *P* = 0.0388, *P* = 0.0279 and *P* = 0.0151, respectively), IL-10 (ANOVA: *F*_(4, 40)_ = 4.129, *P* = 0.0261, *P* = 0.0493 and *P* = 0.0064, respectively) and IFN-γ (ANOVA: *F*_(4, 40)_ = 4.236, *P* = 0.0497, *P* = 0.0035 and *P* = 0.0117, respectively) (Fig. 4b, c, e). However, all the tested doses of rHcADRM1 had no notable effects on secretions of IL-2 (ANOVA: *F*_(4, 40)_ = 0.6883, *P* = 0.4558, *P* = 0.5343, *P* = 0.8819 and *P* = 0.4854, respectively), IL-17A (ANOVA: *F*_(4, 40)_ = 0.8377, *P* = 0.9966, *P* = 0.4641, *P* = 0.9876 and *P* = 0.9967, respectively) and TGF-β1 (ANOVA: *F*_(4, 40)_ = 1.480, *P* = 0.9869, *P* = 0.9952, *P* = 0.9779 and *P* = 0.2874, respectively) production in comparison to the unstimulated group (Fig. 4a, d, f). Collectively, rHcADRM1 stimuli exerted immunomodulation activities on host T cells *via* the alteration of cytokine secretion profiles.

## Discussion

Much of our understanding of parasite immune evasion comes from the sophisticated and highly integrated mechanisms underlying their coexistence with hosts, e.g. parasitic helminths could release ES products or proteins into the host environment to suppress or subvert host immune responses to ensure their survival capabilities [34, 35]. As an individual ES component derived from HcESPs that interacted with host T cells, the *HcADRM1* gene was completely cloned, and the rHcADRM1 protein was successfully obtained in the present study. While ADRM1 proteins are ubiquitously distributed and highly conserved from yeasts to mammals [3, 4], HcADRM1 protein was confirmed to belong to the ADRM1 family, forming a cluster close to *T. circumcincta* orthologue but distant from vertebrate ADRM1 homologues based on the phylogenetic analysis. Consistent with prior identification of rHcADRM1 by sera from infected goats [16], native HcADRM1 protein from HcESPs could be appraised by specific rat anti-HcADRM1 IgG, which in turn validated our preliminary LC-MS/MS data (our unpublished data). Like mammalian ADRM1 as an anticancer target [13], HcADRM1 with outstanding diagnostic utility might be a favourable vaccine candidate for therapeutic prevention against *H. contortus* infection. In addition, we observed enriched cytosolic localizations of native HcADRM1 proteins within the internal cuticle and gut region of adults, probably indicating the active/passive secretions or excretions of HcADRM1 *via* the worm cuticle or gut [35–37]. The *ADRM1* gene is ubiquitously expressed in human tissues with the highest levels in the testis [38], providing a rationale for the higher transcriptional level of the *HcADRM1* gene in male adults rather than female adults of *H. contortus*. Importantly, the amounting expression of HcADRM1 during developmental life-cycle stages demonstrated the crucial role of HcADRM1 in larvae development and survival of *H. contortus*.

Although the existence of ADRM1 is required for cellular proteostasis networks involving transcriptional regulation, cell cycle, apoptosis and immune response, determining the accurate biological roles of ADRM1 individually is complicated due to its functional redundancy shared with RPN1 and RPN10 and a complex structure-function relationship with Uch37 [39–41]. Moreover, to our knowledge, currently there are no data related to the regulation and physiological functions of parasite ADRM1 proteins. Here, we observed that exogenous rHcADRM1 protein bound positively to host T cells *in vitro* and showed cytomembrane and cytoplasmic localizations. Based on previous studies which demonstrated the functional relationships between proteostatic stress and cellular survival [42], we next validated the modulatory impacts of rHcADRM1 on cell viability and apoptosis of host effector cells as potential immunomodulator and external stimuli. Unsurprisingly, the treatments of rHcADRM1 proteins dramatically inhibited T cell viability and induced cell apoptosis. As for the cell apoptosis pathway, initiator caspases like caspase-8 and caspase-9 triggered by apoptotic signals oligomerize and become cleaved, and lead to the activation of downstream targets such as caspase-3 [43]. Subsequently, these effector caspases in turn induce apoptosis *via* cleavage and modifications of target proteins [44]. The oligomerization of death receptors triggers the activation of caspase-8 in extrinsic apoptosis pathway, whereas cytochrome C released from damaged mitochondria initiates caspase-9 activation in intrinsic apoptosis pathway [43, 45]. Intriguingly, rHcADRM1 stimuli notably advanced caspases-8, -9 and -3 transcription, indicating the potential mechanism underlying HcADRM1-induced intrinsic and extrinsic apoptosis of T cells.

For cell apoptosis, proliferation and cell cycle are fundamental and ultimately linked cellular events [46]; we further verified potential immunomodulatory effects of rHcADRM1 on cell proliferation and revealed that rHcADRM1 protein eminently restrained T cell growth *in vitro*. In eukaryotic cells, cell cycles are controlled by the G1/S checkpoint *via* CDK2-cyclin E or CDK4/6-cyclin D kinase compositions, alongside the G2/M checkpoint *via* cyclin B-CDK1 kinase complex [47]. In most cases, the Ub proteasome system is accountable for protein homeostasis and extensively associated with the removal of sabotaged checkpoint proteins to ensure proper timing of cell cycle phase to the next [2]. In this study, our flow cytometry data suggested that rHcADRM1 caused cell cycle stalling at the G1 phase, but not the G2/M phase. Furthermore, the transition through the G1 phase into S phase in T cells was prevented by rHcADRM1 *via* the downregulation of CCNE1 and CDK2 transcriptions and the upregulation of p21 and p27 transcriptions. Collectively, the Ub proteasome pathway, along with nuclear export, is vital for the timely and acute degradation/destruction of all accumulated regulatory proteins in cell division process [48]. Together with Uch37, ADRM1 protein is imperative for the precise cell cycle progress and failure of stable expression of Uch37 and ADRM1 leads to G1 arrest [49]. Therefore, the external stimuli of exogenous rHcADRM1 may hamper the ADRM1-Uch37 interaction in host T cells, disrupt the balance of cell cycle protein degradation and interfere with ADRM1 substrate IκBα signaling along with its downstream effectors [49, 50]. The latter was demonstrated by the transcriptional analysis of IκBα and NF-κB in the present study. Taken together, this could be the mechanistic study of how HcADRM1 targets the regulation of host T cell cycle, apoptosis and proliferation.

Immunomodulation mediated *via* ES proteins of parasitic helminths is generally characterized with a plethora of attributes: blocking pro-inflammatory and Th1 cytokines such as IL-2 and IFN-γ; inducing anti-inflammatory cytokines including IL-10 and TGF-β; modulating Th2 responses like the secretion of IL-4; and regulating Th17 and Treg responses [35, 51]. Consistent with these findings, rHcADRM1 stimuli significantly suppressed the secretion of IL-4, but not IL-2, indicating the critical controls of HcADRM1 on Th2 responses probably *via* the induction of Th2 apoptosis. In addition, our data revealed that IFN-γ and IL-10 secretion was downregulated by rHcADRM1 stimuli. IκBα is one of the physiologic substrates of ADRM1, as well as an essential binding subunit of NF-κB [50, 52]. Thus, the decreased levels of IκBα proteasomal degradation resulting from external rHcADRM1 stimuli may inhibit the NF-κB pathway connected with inflammation reactions and cell growth, thereby may in turn inhibit IFN-γ, IL-4 and IL-10 secretions [53, 54]. Taken together, we hereby identified HcADRM1 as an immunomodulator at the parasite-host interface *via* the inhibition of T cell survival and growth. However, due to the absence of available goat immune reagents, we only validated the transcription levels of several key molecules involving apoptosis, proliferation and cell cycle in this study. More detailed mechanistic networks at the protein level, alongside associated pathways, merit further investigation. Clearly, future exploration is indispensable to address the potential role of HcADRM1 for prophylactic therapy in anti-*H. contortus* vaccine development.

## Conclusions

We identified and characterized a novel ADRM1 orthologue with immunomodulatory utility derived from HcESPs. Employing the bioinformatic approaches integrated with immunological bioassays, we demonstrated that HcADRM1 protein is expressed in all developmental stages of *H. contortus* and could interact with host key effector cells and interfere with a plethora of cellular events *via* repression of T cell viability and proliferation, facilitation of T cell apoptosis, stalling of dynamic T cell cycle, disruption of pathway signaling and alteration of cytokine production profiles. To our knowledge, this is a proteomic-guided comprehensive investigation for parasite orthologues of the ADRM1 family. The results of this study may improve our understanding of ADRM1 proteins from parasitic nematodes and continue to illustrate the diverse range of immunomodulatory activities of ES proteins.

## Supplementary information

**Additional file 1: Figure S1.** Goat T cell sorting by MACS. The purity of isolated T cells was validated *via* flow cytometry to be above 95% as indicated before (**a**) and after (**b**) MACS sorting.

**Additional file 2: Table S1.** Primer sequences for HcADRM1 transcription analysis. **Table S2.** Primer sequences for the transcription analysis of apoptosis and cell cycle.

## Data Availability

The datasets supporting the conclusions of this article are included within the article and its additional files.

## Abbreviations

Ub: ubiquitin
RP: regulatory particle
LC-MS/MS: liquid chromatography-tandem mass spectrometry
ADRM1: adhesion-regulating molecule 1
IL: interleukin
TGF: transforming growth factor
IFN: interferon
PBMCs: peripheral blood mononuclear cells
ES: excretory-secretory
HcESPs: *Haemonchus contortus* ES proteins
IκBα: NF-κB inhibitor alpha
CDK: cyclin-dependent kinase
CCK-8: cell counting kit-8
ConA: concanavalin A
7-AAD: 7-aminoactinomycin D
EdU: 5-ethynyl-2′-deoxyuridine
NF-κB: nuclear factor kappa B
pAb: polyclonal antibody
