# Supplementary figures and images for: Unveiling the immunomodulatory properties of Haemonchus contortus adhesion regulating molecule 1 interacting with goat T cells
Source: Parasit Vectors. 2020 Aug 18;13:424. doi: 10.1186/s13071-020-04297-7 (PMC7432459; doi:10.1186/s13071-020-04297-7)

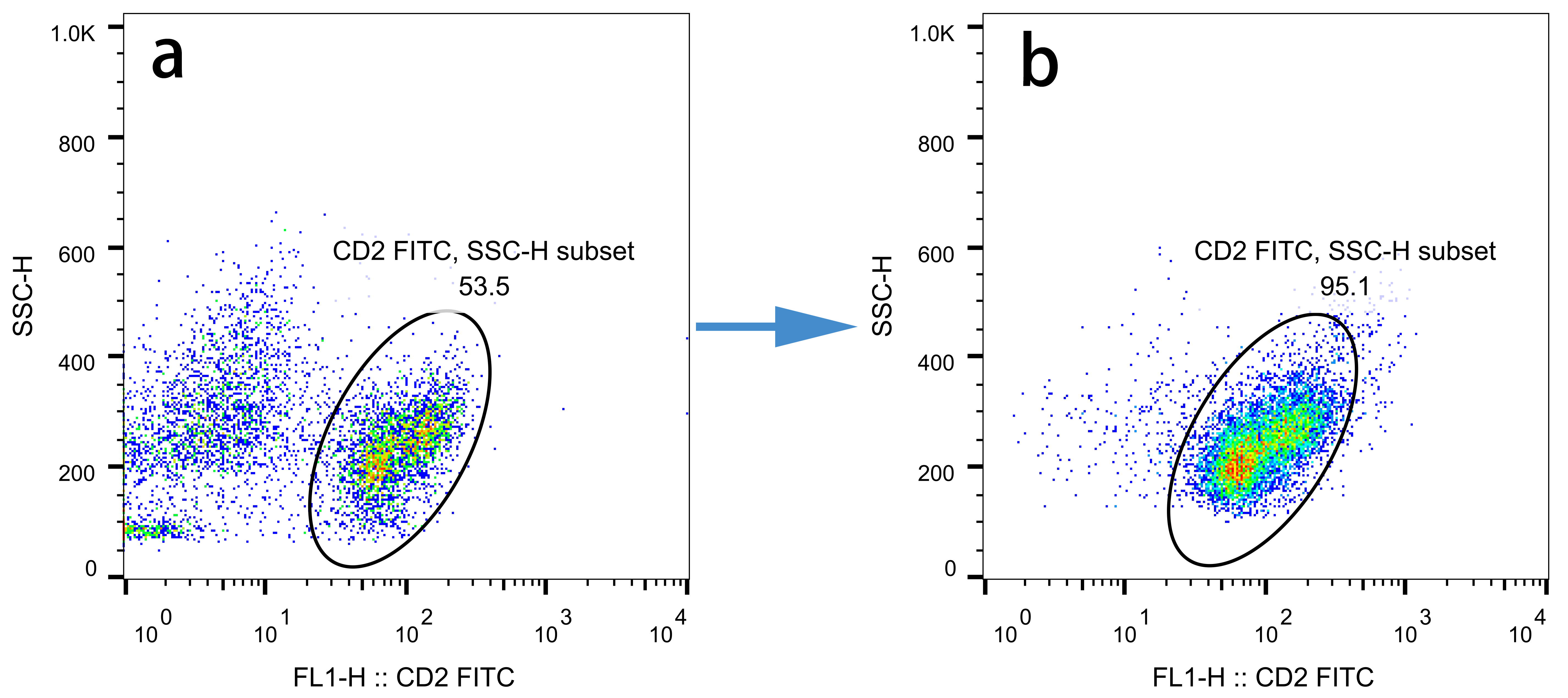

Supplement: Supplementary file 1 — Additional file 1: Figure S1. Goat T cell sorting by MACS. The purity of isolated T cells was validated via flow cytometry to be above 95% as indicated before (a) and after (b) MACS sorting. [file 13071_2020_4297_MOESM1_ESM.tif]
