# Supplementary material for: Unveiling the immunomodulatory properties of Haemonchus contortus adhesion regulating molecule 1 interacting with goat T cells
Source: Parasit Vectors. 2020 Aug 18;13:424. doi: 10.1186/s13071-020-04297-7 (PMC7432459; doi:10.1186/s13071-020-04297-7)
Supplement: Supplementary file 2 — Additional file 2: Table S1. Primer sequences for HcADRM1 transcription analysis. Table S2. Primer sequences for the transcription analysis of apoptosis and cell cycle. [file 13071_2020_4297_MOESM2_ESM.docx]

**Additional file 2: Table S1. Primer sequences for HcADRM1 transcription analysis**

| **Gene Name** | **Primer Sequence (5’-3’)** | **Reference** | **Size (bp)** |
| --- | --- | --- | --- |
| β-Tubulin | F: TGCTATGTTCCGTGGTCGTATG  R: CGGCAGTCTTAACGTTGTTTGG | [24] | 116 |
| HcADRM1 | F: CTCAATCACATGCCCGTAGAA  R: TGTTGGCAGCTTGTCTGTAA | CDL94593.1 | 117 |

**Additional file 2: Table S2. Primer sequences for the transcription analysis of apoptosis and cell cycle**.

| **Gene Name** | **Primer Sequence (5’-3’)** | **Reference** | **Size (bp)** |
| --- | --- | --- | --- |
| beta-actin | F: CACCACACCTTCTACAAC  R: TCTGGGTCATCTTCTCAC | [29] | 106 |
| Caspase 3 | F: CATTATTCAGGCCTGCCGAG  R: CTCGAGCTTGTGAGCGTACT | [30] | 220 |
| Caspase 8 | F: TTAGCATAGCACGGGAGCAG  R: GTCAGCTCATAGATGGGGGC | [30] | 280 |
| Caspase 9 | F: GGGAAATGCTGATCTGGCCT  R: CAGCCGTGAGAGAGGATGAC | [30] | 279 |
| CCND1 | F: GGTCCTGGTGAACAAACTC  R: TTGCGGATGATCTGCTT | [31] | 114 |
| CDK4 | F: CGTTGGCTGTATCTTTGC  R: GATTCGCTTGTGTGGGTT | [31] | 256 |
| CDK6 | F: AGAGTGATTGCAGCTTTATGTCCA  R: TGCCCAGGTTGCTCACTTC | [31] | 157 |
| CCNE1 | F: GGGACAAGCACCTTATGCAAC  R: GTGTTGCCATATACCGATCAAAGA | [31] | 153 |
| CDK2 | F: CTGCACCGAGACCTTAAACCTCA  R: GCTCGGTACCACAGAGTCACCA | [31] | 140 |
| p21 | F: CTAAGTGGGCAAATATGGGTCTGG  R: CAGGATGCTACAGGAGCTGGAAG | [32] | 107 |
| p27 | F: AAACCCAGAGGACACGCATT  R: GGCAGGTCGCTTCCTTATCC | [32] | 100 |
| IκBα | F: GGTGAAGGAGCTGCGAGAG  R: GCTCACAGGCAAGGTGTAGG | [33] | 326 |
| NF-κB | F: CTTCCATCCTGGAACCACTAAA  R: ACCTCTCTGTCGTCACTCTT | [33] | 108 |
